# Application of Machine Learning Models in Predicting Non-Alcoholic Fatty Liver Disease Among Inactive Chronic Hepatitis B Patients: A Cross-Sectional Analysis

**DOI:** 10.3390/jcm14145042

**Published:** 2025-07-16

**Authors:** Abdullah M. Al-Alawi, Amna S. Al-Balushi, Halima H. Al-Shuaili, Dalia A. Mahmood, Said A. Al-Busafi

**Affiliations:** 1 Department of Medicine, Sultan Qaboos University Hospital, Muscat 123, Oman; 2 Internal Medicine Program, Oman Medical Specialty Board, Muscat 130, Oman; 3 Department of Medicine, Armed Forces Hospital, Muscat 112, Oman; 4 General Practice, Oman International Hospital, Muscat 333, Oman; 5 Department of Medicine, College of Medicine and Health Sciences, Sultan Qaboos University, Muscat 123, Oman

**Keywords:** NAFLD, chronic hepatitis B, machine learning, predictive features, XGBoost

## Abstract

**Background/Objectives**: Non-alcoholic fatty liver disease (NAFLD) represents significant health challenges, especially among patients with chronic hepatitis B (CHB). This study uses machine learning models to predict NAFLD in patients with inactive CHB. It builds on previous research by employing classification algorithms to analyze demographic, clinical, and laboratory data to identify NAFLD predictors. **Methods**: A single-center cross-sectional study was conducted, including 450 inactive CHB patients from Sultan Qaboos University Hospital. Five ML models were developed: Logistic Regression, Random Forest, Extreme Gradient Boosting (XGBoost), Support Vector Machine (SVM), and Multi-Layer Perceptron (MLP). **Results**: The prevalence of NAFLD was 50.22%. Among the machine learning models, Random Forest achieved the highest performance with an ROC AUC of 0.983 (95% CI: 0.952–0.999), followed by XGBoost at 0.977 (95% CI: 0.938–0.999) and MLP at 0.963 (95% CI: 0.915–0.995). SVM also showed strong performance with an AUC of 0.949 (95% CI: 0.897–0.985), while Logistic Regression demonstrated comparatively lower discrimination with an AUC of 0.886 (95% CI: 0.799–0.952). Key predictive features identified included platelet count, low-density lipoprotein (LDL), hemoglobin, and alanine aminotransferase (ALT). Logistic Regression highlighted platelet count as the most significant negative predictor, while LDL and ALT were positive contributors. **Conclusions**: This study shows the utility of ML in improving the identification and management of NAFLD in CHB patients, enabling targeted interventions. Future research should expand on these findings, integrating genetic and lifestyle factors to enhance predictive accuracy across diverse populations.

## 1. Introduction

The hepatitis B virus (HBV) is a significant global health issue, affecting hundreds of millions worldwide. Recent estimates show that chronic HBV infection impacts approximately 296 million people globally [1,2]. Specific populations show varying prevalence rates: among pregnant women, the prevalence is 4.8% (95% CI: 3.8–5.8%) [3], among people living with HIV, it is about 10–11% [4], and among patients with tuberculosis, the seroprevalence is 5.8% (95% CI: 5.0–6.8%) [5]. Regionally, the highest HBV prevalence rates are in the WHO African Region and the Western Pacific. Although the global prevalence and incidence of HBV-related liver disease have decreased from 2000 to 2021, some areas, particularly Europe and the Americas, have seen an increase in HBV-related liver cancer prevalence [2,5].

Inactive chronic hepatitis B (CHB) is marked by long-term presence of HBsAg, normal transaminase levels, HBeAg negativity, anti-HBe positivity, low or undetectable HBV DNA, and minimal liver inflammation [6]. Inactive carriers have a favorable prognosis with a low risk of severe liver disease, but there is a risk of reactivation leading to liver damage. Low HBV DNA (<2000 IU/mL) and HBsAg (<1000 IU/mL) levels indicate inactivity and reduced disease progression risk [7]. Regular monitoring is crucial to detect potential reactivation or disease progression. Some patients enter an “indeterminate phase,” posing higher hepatocellular carcinoma (HCC) risks compared to truly inactive carriers [8].

Non-alcoholic fatty liver disease (NAFLD) involves fat accumulation in liver cells and can advance to non-alcoholic steatohepatitis (NASH), fibrosis, cirrhosis, and HCC [9,10]. Among those with NAFLD, about 24% develop NASH [11].

NAFLD is commonly associated with metabolic syndrome, obesity, and diabetes and can lead to liver fibrosis and cirrhosis. It disrupts liver cell function through dysregulated fatty acid metabolism and lipotoxicity, impacting HBV replication and immune response [12]. Studies show that NAFLD is common among patients with CHB, with prevalence rates ranging from 21% to 37.4% [11,13,14]. NAFLD is becoming more prevalent in patients with CHB due to rising obesity and metabolic syndrome rates [12,15]. NASH, a severe form of NAFLD, independently predicts significant fibrosis in CHB patients, potentially increasing HCC risk. In CHB patients with NAFLD, significant fibrosis (stage ≥ F2) occurs in approximately 10% to 21% of cases [14]. CHB can also contribute to metabolic changes that exacerbate NAFLD and liver inflammation. Clinically, it is important to routinely assess NAFLD in CHB patients, as it heavily influences disease progression and treatment outcomes. Changes in NAFLD status may influence the effectiveness of antiviral therapy in patients with CHB, particularly regarding biochemical response and fibrosis improvement, although current evidence primarily supports this association in those receiving entecavir (ETV), while data for tenofovir (TDF) remain limited and inconclusive [11,16].

Increased waist circumference and body mass index (BMI) are significant risk factors for NAFLD in patients with CHB, alongside elevated triglycerides and low-density lipoprotein (LDL) levels [17,18]. A history of diabetes and higher insulin resistance, as measured by HOMA-IR scores, are also strong predictors [17]. Among viral factors, different types of HBV infections affect NAFLD prevalence, with HBeAg-negative hepatitis and immunotolerant phases showing lower rates compared to past CHB infections [15]. Long-term antiviral therapy for CHB is linked to an increased risk of NAFLD. Other associated factors include hypertension, elevated serum uric acid, and higher alkaline phosphatase (ALP) levels [17].

A recent study involving patients with inactive CHB found a 47.8% prevalence of NAFLD. Key risk factors for NAFLD included type 2 diabetes, high LDL, high hemoglobin, low platelet counts, and normal alpha-fetoprotein levels. NAFLD was significantly associated with increased fibrosis, with 10.5% of those with NAFLD having significant fibrosis compared to 1.4% without NAFLD [10].

Machine learning enhances disease diagnosis by using advanced algorithms to analyze complex biomedical data, resulting in more accurate, timely, and personalized outcomes. ML improves diagnostic accuracy by incorporating causal reasoning, which distinguishes correlation from causation, and models like support vector machine (SVM) and neural networks demonstrate high sensitivity and specificity in diagnosing diseases [19,20]. It aids in the early detection of neurodegenerative and chronic diseases by integrating data sources for early marker identification and predictive modeling [21]. ML also handles complex and rare diseases by using diverse data types and algorithms to diagnose rare conditions and assist in infectious disease management, including predicting treatment responses and antibiotic resistance [22]. Methodologies involve both supervised and unsupervised learning, with Bayesian methods providing a probabilistic framework for dealing with uncertainty. ML integrates multimodal data to enhance accuracy and uses big data to refine disease models and personalize treatment strategies [18,23,24].

Very few studies have suggested that machine learning models are highly effective in predicting and screening for NAFLD. Extreme gradient boosting (XGBoost), for instance, has demonstrated high accuracy (0.880), precision (0.801), recall (0.894), F1-score (0.882), and AUC (0.951) in large-scale population screenings, using covariates like BMI, age, waist circumference, gender, and type 2 diabetes [25]. ML approaches using blood biomarkers can predict NAFLD and distinguish it from NASH, offering a cost-effective method by ranking important features [25,26]. For identifying NAFLD stages, ML models such as logistic regression, random forest, and artificial neural networks outperform traditional non-invasive tests like FibroScan and FIB-4 in detecting liver fibrosis and cirrhosis, providing higher accuracy and AUC values [27].

In view of the high prevalence of NAFLD among patients with CHB infection, and the potential role of ML and AI in detecting early disease, as well as their promising prospects in the management of chronic liver disease, we recognize the low number of studies on the role of AI and ML in diagnosing NAFLD. This study utilizes ML models to predict the presence of NAFLD among patients with inactive CHB, expanding on previous research using traditional statistical methods [10]. This study employs classification algorithms to analyze demographic, clinical, and laboratory data to identify predictors of NAFLD.

## 2. Materials and Methods

The detailed methods and materials were described previously [10]. This study utilized a cross-sectional design based on retrospective data. Each patient was assessed at a single time point using clinical records from 2010 to 2021. NAFLD diagnosis was based on ultrasound findings available at the time of routine CHB follow-up visits, and no longitudinal follow-up was conducted to capture incident NAFLD or temporal progression.

Patients lacking essential data like laboratory or imaging results were excluded. Data were collected from electronic medical records via SQUH’s TrakCare system (TrakCare, InterSystems Corp., Cambridge, MA, USA). Baseline information included demographics (age, gender, weight, height, and BMI) and medical history (hypertension, type 2 diabetes, dyslipidemia, and metabolic syndrome). Laboratory data covered hepatitis B markers, HBV viral load, alpha-fetoprotein, liver enzymes, hemoglobin, platelet count, lipids, and glycated hemoglobin. Blood pressure readings were also recorded. Routine screening for HCC was conducted every six months using liver ultrasounds paired with two-dimensional shear wave elastography, utilizing the GE LOGIQ E9 XDclear 2.0 machine (GE Healthcare, Chicago, IL, USA). These screenings were evaluated for indications of NAFLD and cirrhosis.

### 2.1. Definitions

Inactive CHB was defined according to the European Association for the Study of the Liver (EASL) guidelines. Criteria involved positive serum anti-HBeAg, HBV DNA levels undetectable or below 2000 IU/mL via PCR testing, and normal ALT levels. Some patients with HBV DNA levels between 2000 and 20,000 IU/mL were included if their ALT levels remained normal and if they showed low fibrosis [22].

NAFLD was diagnosed using ultrasound findings according to the criteria set by the American Association for the Study of Liver Diseases (AASLD). These criteria require evidence of steatosis through imaging or histology and the exclusion of secondary causes, such as excessive alcohol use, other viral hepatitis, or steroid use [28].

The FIB-4 index is a non-invasive scoring system used to estimate liver fibrosis based on routine laboratory tests. It is calculated using the following formula: FIB-4 = (Age × AST)/(Platelet count × √ALT). This score is particularly useful in chronic liver disease to stratify patients into low, intermediate, or high risk of advanced fibrosis. In this study, the FIB-4 index was calculated for all patients with available data and included as a continuous variable in both descriptive analysis and predictive modeling [10].

### 2.2. Ethics

This study received ethical approval from the institutional Medical Research and Ethics Committee of the College of Medicine and Health Sciences at Sultan Qaboos University. All study procedures were conducted in accordance with the principles of the revised Helsinki Declaration.

### 2.3. Statistical Analysis

Missing continuous variable values were imputed with the median to reduce outlier effects, while categorical variables used the mode. Continuous variables were standardized with StandardScaler to achieve a mean of 0 and a standard deviation of 1 for better model convergence and interpretability. Before building a predictive model, chi-square tests and *t*-tests are used for preliminary feature selection. These tests identify statistically significant variables, reducing dimensionality and eliminating irrelevant features to enhance model interpretability and performance. The chi-square test assesses associations for categorical variables, and the t-test evaluates differences for continuous variables. Variables with *p* < 0.2 were included in the model.

Five ML models—Logistic Regression, Random Forest, XGBoost, SVM, and Multi-Layer Perceptron (MLP) Neural Network—were developed to predict NAFLD. The dataset was split into training and testing sets in an 80–20 ratio. Models were trained on the training set, and their performance was evaluated on the testing set using metrics such as accuracy, precision, recall (sensitivity), and the F1-score to balance precision and recall. We identified the ten most influential features contributing to NAFLD in each model and used a logistic regression model to determine the direction of associations with NAFLD.

## 3. Results

The study included 450 patients. Data were gathered on 25 continuous variables (Table 1) and 18 categorical variables (Table 2). Only four patients were positive for HBsAb, despite meeting all criteria for inactive chronic hepatitis B (CHB) infection, including persistent HBsAg positivity and low or undetectable HBV DNA for ≥6 months. This finding may reflect partial seroconversion, immune recovery, or assay variability.

The age group of 40–60 years was the most represented, comprising 63.1% of participants, and 54% were male. The prevalence of NAFLD was 50.22% (95% CI: 45.62–54.82). Common comorbidities included dyslipidemia (16.9%), diabetes mellitus (15.3%), and hypertension (12.9%). After deploying chi-square tests and *t*-tests for preliminary feature selection, 18 features were included out of 42, comprising 13 continuous and 5 categorical variables (Table 3). In this study, five machine learning models—Logistic Regression, Random Forest, XGBoost, Support Vector Machine (SVM), and Multi-Layer Perceptron (MLP) Neural Network—were developed and compared to identify the best predictive model for NAFLD. The Random Forest model demonstrated the highest performance, with an ROC AUC score of 0.983 (95% CI: 0.952–0.999), followed closely by XGBoost [0.977 (95% CI: 0.938–0.999)] and MLP [0.963 (95% CI: 0.915–0.995)]. SVM also performed well with an AUC of 0.949 (95% CI: 0.897–0.985). Logistic Regression showed comparatively lower, yet acceptable, performance with an AUC of 0.886 (95% CI: 0.799–0.952), indicating that all models demonstrated good discriminatory ability, with tree-based methods and neural networks outperforming linear models. (Table 4).

Feature importance analysis showed that platelet count, LDL, hemoglobin, and alanine aminotransferase (ALT) consistently emerged as key predictors across Logistic Regression, Random Forest, XGBoost, and SVM. Other significant features include hemoglobin A1C (HbA1C), fasting blood sugar, alpha-fetoprotein (AFP), and systolic blood pressure (BP). Gender and age groups also played critical roles, particularly in SVM. The antibody to hepatitis B Surface Antigen (Anti-HBs) exhibited the highest importance (Table 5).

**Figure 1:**
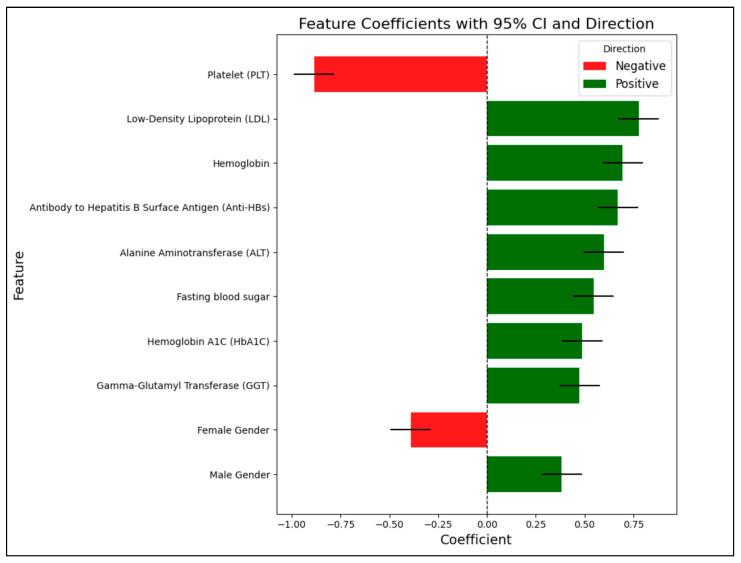
Feature importance and direction in logistic regression for NAFLD prediction.

Furthermore, Logistic Regression identified the platelet count as the most significant feature, with a negative association indicating that higher platelet levels reduce the likelihood of NAFLD. Positive contributors included metabolic and hepatic indicators such as LDL, hemoglobin, ALT, fasting blood sugar, and hemoglobin A1C (HbA1C). Gender differences are evident, with male gender positively associated with NAFLD (Table 6, Figure 1).

## 4. Discussion

This study used advanced ML techniques to predict NAFLD in a specific patient population, demonstrating high predictive accuracy, especially with XGBoost. It identified significant predictors like platelet count, LDL, and ALT, adding to clinical relevance by bridging ML applications with routine practice.

The prevalence of NAFLD among inactive CHB patients was 50.22%. This finding is higher than previously reported prevalence rates, which have ranged from 21% to 37.4% [11,13,14]. This could be explained by the higher prevalence of risk factors, including common comorbidities such as dyslipidemia and diabetes mellitus. This study corroborates earlier research, showing that key metabolic markers such as ALT, LDL, and HbA1C are strong positive predictors of NAFLD, emphasizing the importance of monitoring these factors in routine clinical practice [12,13,17].

In this study, five machine learning models achieved high sensitivity and specificity, with XGBoost achieving the highest ROC AUC score of 0.9580, followed by Random Forest and Multi-Layer Perceptron, while Logistic Regression and SVM showed moderate performance. This emphasizes the utility of ML in identifying high-risk individuals. This approach complements traditional clinical methods, enhancing diagnostic precision and enabling personalized intervention strategies.

ML models can enhance treatment strategies for chronic liver disease. An ML-based tool was developed to recommend therapies like stereotactic body radiotherapy (SBRT) or radiofrequency ablation (RFA) for patients with unresectable HCC, improving progression-free survival when followed. Additionally, machine learning can predict complications after liver transplantation, such as new-onset diabetes mellitus, with a random forest model achieving 79.5% accuracy, aiding in early intervention and improved post-transplant management [29,30].

Preventing NAFLD requires a comprehensive approach that includes dietary modifications, such as increasing polyphenols and reducing free sugars, adopting healthy eating patterns like the Mediterranean diet, and incorporating specific bioactive compounds like green tea and garlic [31,32]. Lifestyle changes, particularly weight loss and regular exercise, are also essential. These strategies collectively help reduce liver fat, oxidative stress, and inflammation, thereby preventing the onset and progression of NAFLD [31,32].

Logistic Regression identified the platelet count as the most significant feature, which was negatively associated with NAFLD likelihood. Positive contributors included LDL, hemoglobin, ALT, fasting blood sugar, and HbA1C.

Gender differences in NAFLD show males positively and females negatively associated with the disease, highlighting complex risk factors across metabolic, hematologic, and demographic domains. Sex at birth influences NAFLD development and progression, with distinct metabolic signatures in males and females [33]. In adolescents, male sex is linked to higher liver fat and NAFLD risk [33]. Metabolomic profiles reveal men with NAFLD have increased amino acids, while women show different patterns, indicating sex-specific pathways [34]. Animal models suggest males may develop more pronounced cardiac dysfunction, whereas females may experience different cardiometabolic impairments, underscoring sex-dependent effects [35].

The integration of ML tools in clinical workflows for inactive CHB patients can transform the management of NAFLD by enabling early detection and targeted interventions. Future studies should explore the scalability of these models across diverse populations and healthcare settings.

The single-center nature of this study may limit the generalizability of its findings to broader populations. Additionally, although ultrasound is a practical and widely available tool for detecting NAFLD, it may underdiagnose milder cases compared to more sensitive imaging modalities such as MRI. Longitudinal studies are warranted to better characterize the progression of NAFLD among patients with chronic hepatitis B (CHB).

It is also important to note that the term non-alcoholic fatty liver disease (NAFLD) was used throughout this study, as data collection and diagnostic definitions were completed prior to the 2023 introduction of the updated nomenclature, which is metabolic dysfunction-associated steatotic liver disease (MASLD) [36]. While the terminology has since evolved, our study adhered to the criteria in place at the time. Future research should adopt the MASLD framework to align with the current consensus and enhance cross-study comparability. Furthermore, refining machine learning models by integrating additional predictors—such as genetic markers, lifestyle data, and advanced imaging—may improve predictive performance and clinical utility.

## 5. Conclusions

This study highlights a 50.22% prevalence of NAFLD among inactive CHB patients and identifies critical predictors through ML models. XGBoost outperformed other models, demonstrating the potential of ML in early NAFLD detection. Incorporating ML tools into clinical workflows can transform the management of NAFLD, enabling early intervention and personalized care strategies. Further multicenter studies are warranted to validate these findings.

## Figures and Tables

**Table 1:** Detailed descriptive statistics of relevant continuous variables (n = 450).

Variable (n = 450 Patients)	Median/Mean (IQR/SD)
Body Mass Index (BMI) [kg/m^2^]	28.7 ± 4.6
Weight [Kg]	77.1 (69.0–86.375)
Height [cm]	161.8 ± 14.7
Hepatitis B Virus Polymerase Chain Reaction (HBV PCR) [viral load]	90.0 (32.0–344.25)
Alpha-Fetoprotein (AFP) [ng/mL]	3.0 (2.0–5.0)
Gamma-Glutamyl Transferase (GGT) [U/L]	23.49 ± 6.3
Alanine Transaminase (ALT) [U/L]	18.5 (15.0–26.0)
Alkaline Phosphatase (ALP) [U/L]	69.0 (60.0–78.75)
Aspartate Aminotransferase (AST) [U/L]	21.0 (17.0–25.0)
Bilirubin [µmol/L]	7.0 (6.0–9.0)
Hemoglobin [g/dL]	12.7 (11.6–13.775)
White Blood Cell Count (WBC) [×10^9^/L]	5.3 (4.3–6.6)
Platelet Count [×10^9^/L]	274.0 (222.0–333.0)
Fasting Blood Sugar [mmol/L]	5.7 ± 1.6
Random Blood Sugar [mmol/L]	6.7 (6.7–6.7)
Hemoglobin A1c (HbA1C) [%]	5.5 (5.4–5.5)
Systolic Blood Pressure (BP) [mmHg]	129.0 (125.0–133.0)
Diastolic Blood Pressure (BP) [mmHg]	76.0 (72.0–79.75)
Fibrosis-4 Index (FIB-4)	0.65 (0.51–0.85)
Albumin [g/L]	43.0 (40.0–46.0)
Total Cholesterol [mmol/L]	4.6 (4.125–5.1)
Triglyceride [mmol/L]	1.3 (1.2–1.6)
Low-Density Lipoprotein (LDL) [mmol/L]	2.6 (2.3–3.0)
High-Density Lipoprotein (HDL) [mmol/L]	1.2 (1.1–1.3)

**Table 2:** Detailed descriptive statistics of relevant categorical variables (n = 450).

Variable	Count (n, %)
Anti-Hbe	450 (100.0%)
HbsAg	450 (100.0%)
Anti-HBc	450 (100.0%)
Age Group (40–60 years)	284 (63.1%)
Sex (Male)	243 (54.0%)
Non-Alcoholic Fatty Liver Disease (NAFLD)	226 (50.2%)
Age Group (18–39 years)	139 (30.9%)
Regular Medications (OHA, Insulin, Statins, Anti-HTN meds, Aspirin, CVD-related meds)	109 (24.2%)
Dyslipidemia	76 (16.9%)
Diabetes Mellitus (DM)	69 (15.3%)
Metabolic Syndrome	68 (15.1%)
Hypertension (HTN)	58 (12.9%)
Age Group (>60 years)	27 (6.0%)
Cardiovascular Disease	24 (5.3%)
Anti-HBs	4 (0.9%)
Smoking	3 (0.7%)

**Table 3:** Relevant features with *p*-value < 0.2 included in model development.

Feature
Categorical variable
Antibody to Hepatitis B Surface Antigen (Anti-HBs)
Age Group (18–39, 40–60, >60)–40–60
Age Group (18–39, 40–60, >60)–18–39
Male Gender
Female Gender
Non-Alcoholic Fatty Liver Disease (NAFLD)
Continuous variable
Body Mass Index (BMI)
Weight (Kg)
Alpha-Fetoprotein (AFP)
Gamma-Glutamyl Transferase (GGT)
Alanine Aminotransferase (ALT)
Low-Density Lipoprotein (LDL)
Hemoglobin
White Blood Cell Count (WBC)
Platelet Count
Fasting Blood Sugar
Hemoglobin A1C (HbA1C)
Systolic Blood Pressure (Systolic BP)
Fibrosis-4 (FIB-4) Index

**Table 4:** Performance metrics of machine learning models for NAFLD prediction.

Model	Accuracy (Proportion Correct)	Precision (Positive Predictive Value)	Recall (Sensitivity)	F1-Score (Harmonic Mean of Precision and Recall)	ROC AUC Score (95% CI)
Logistic Regression	0.7889	0.7708	0.8222	0.7957	0.886 (0.799–0.952)
Random Forest	0.8444	0.8163	0.8889	0.8511	0.983 (0.952–0.999)
Extreme Gradient Boosting (XGBoost)	0.8667	0.8511	0.8889	0.8696	0.977 (0.938–0.999)
Support Vector Machine (SVM)	0.8000	0.7755	0.8444	0.8085	0.949 (0.897–0.985)
Multi-Layer Perceptron (MLP)	0.8667	0.8367	0.9111	0.8723	0.963 (0.915–0.995

**Table 5:** Top 10 contributing features across machine learning models for NAFLD prediction.

Model	Feature	Importance
Logistic Regression	Platelet (PLT)	0.884571
	Low-Density Lipoprotein (LDL)	0.777531
	Hemoglobin	0.694546
	Antibody to Hepatitis B Surface Antigen (Anti-HBs)	0.670535
	Alanine Aminotransferase (ALT)	0.597981
	Fasting Blood Sugar	0.545185
	Hemoglobin A1C (HbA1C)	0.487604
	Gamma-Glutamyl Transferase (GGT)	0.474233
	Female Gender	0.391136
	Male Gender	0.382072
Random Forest	Systolic Blood Pressure (BP)	0.148352
	Alpha-Fetoprotein (AFP)	0.125458
	Low-Density Lipoprotein (LDL)	0.109997
	Alanine Aminotransferase (ALT)	0.106698
	Hemoglobin	0.098780
	Platelet (PLT)	0.082542
	Hemoglobin A1C (HbA1C)	0.063411
	Weight (Kg)	0.062952
	Fasting Blood Sugar	0.053689
	Fibrosis-4 (FIB-4) Index	0.045929
XGBoost	Alpha-Fetoprotein (AFP)	0.188119
	Systolic Blood Pressure (BP)	0.141393
	Hemoglobin	0.096793
	Alanine Aminotransferase (ALT)	0.095268
	Low-Density Lipoprotein (LDL)	0.086143
	Hemoglobin A1C (HbA1C)	0.073799
	Fasting Blood Sugar	0.061145
	Platelet (PLT)	0.057581
	Fibrosis-4 (FIB-4) Index	0.045751
	Weight (Kg)	0.034774
SVM	Antibody to Hepatitis B Surface Antigen (Anti-HBs)	1.000000
	Platelet	0.725569
	Hemoglobin	0.643911
	Low-Density Lipoprotein (LDL)	0.556026
	Alanine Aminotransferase (ALT)	0.491436
	HbA1C	0.475327
	Age Group (18–39)	0.326932
	Male Gender	0.298697
	Female Gender	0.298697
	Age Group (40–60)	0.279856

**Table 6:** Feature importance and direction in logistic regression for NAFLD prediction.

Feature	Coefficient	Importance	Direction
Platelet (PLT)	−0.884571	0.884571	Negative
Low-Density Lipoprotein (LDL)	0.777531	0.777531	Positive
Hemoglobin	0.694546	0.694546	Positive
Antibody to Hepatitis B Surface Antigen (Anti-HBs)	0.670535	0.670535	Positive
Alanine Aminotransferase (ALT)	0.597981	0.597981	Positive
Fasting Blood Sugar	0.545185	0.545185	Positive
Hemoglobin A1C (HbA1C)	0.487604	0.487604	Positive
Gamma-Glutamyl Transferase (GGT)	0.474233	0.474233	Positive
Female Gender	−0.391136	0.391136	Negative
Male Gender	0.382072	0.382072	Positive

## Data Availability

The original contributions presented in this study are included in the article. Further inquiries can be directed to the corresponding author.

## Abbreviations

NAFLD	Non-Alcoholic Fatty Liver Disease
CHB	Chronic Hepatitis B
ML	Machine Learning
XGBoost	Extreme Gradient Boosting
SVM	Support Vector Machine
MLP	Multi-Layer Perceptron
ROC AUC	Receiver Operating Characteristic Area Under the Curve
LDL	Low-Density Lipoprotein
ALT	Alanine Aminotransferase
